# How should we manage information needs, family anxiety, depression, and breathlessness for those affected by advanced disease: development of a Clinical Decision Support Tool using a Delphi design

**DOI:** 10.1186/s12916-015-0449-6

**Published:** 2015-10-13

**Authors:** Liesbeth M. van Vliet, Richard Harding, Claudia Bausewein, Sheila Payne, Irene J. Higginson, on behalf of EUROIMPACT

**Affiliations:** Department of Palliative Care, Policy and Rehabilitation, Cicely Saunders Institute, King’s College London, Bessemer Road, London, SE5 9PJ UK; Department of Palliative Medicine, Munich University Hospital, Munich, Germany; International Observatory on End of Life Care, Division of Health Research, Lancaster University, Lancaster, UK

**Keywords:** Clinical decision support tools, Delphi studies, Implementation, Palliative care, Palliative care outcome scale (POS), Patient reported outcome measures

## Abstract

**Background:**

Clinicians request guidance to aid the routine use and interpretation of Patient Reported Outcome Measures (PROMs), but tools are lacking. We aimed to develop a Clinical Decision Support Tool (CDST) focused on information needs, family anxiety, depression, and breathlessness (measured using the Palliative care Outcome Scale (POS)) and related PROM implementation guidance.

**Methods:**

We drafted recommendations based on findings from systematic literature searches. In a modified online Delphi study, 38 experts from 12 countries with different professional backgrounds, including four patient/carer representatives, were invited to rate the appropriateness of these recommendations for problems of varying severity in the CDST. The quality of evidence was added for each recommendation, and the final draft CDST reappraised by the experts. The accompanying implementation guidance was built on data from literature scoping with expert revision (n = 11 invited experts).

**Results:**

The systematic literature searches identified over 560 potential references, of which 43 met the inclusion criteria. Two Delphi rounds (response rate 66 % and 62 %; n = 25 and 23) found that good patient care, psychosocial support and empathy, and open communication were central to supporting patients and families affected by all POS concerns as a core requirement. Assessment was recommended for increasing problems (i.e. scores), followed by non-pharmacological interventions and for breathlessness and depression, pharmacological interventions. Accompanying PROM implementation guidance was built based on the 8-step International Society for Quality of Life Research framework, as revised by nine (response rate 82 %) experts.

**Conclusions:**

This CDST provides a straightforward guide to help support clinical care and improve evidence-based outcomes for patients with progressive illness and their families, addressing four areas of clinical uncertainty. Recommendations should be used flexibly, alongside skilled individual clinical assessment and knowledge, taking into account patients’ and families’ individual preferences, circumstances, and resources. The CDST is provided with accompanying implementation guidance to facilitate PROM use and is ready for further development and evaluation.

**Electronic supplementary material:**

The online version of this article (doi:10.1186/s12916-015-0449-6) contains supplementary material, which is available to authorized users.

## Background

Providing optimal care to patients facing life-threatening and progressing illnesses requires a focus on patients’ and their family caregivers’ physical, psychological, emotional, and spiritual needs [1]. It is not solely a task of experts in palliative care. The number of people affected by chronic and progressive illness is escalating, fueled by population ageing and improved life expectancy [2]. While specialist palliative care services, such as hospices and home care teams, provide an extra layer of support for those with the most complex needs, patients and their families with progressive and life threatening illness come into contact with health and social care workers in all settings – primary, secondary, and tertiary. Providing holistic care for seriously ill patients and their families is now a central component of health care [3]. This can, however, be daunting for many doctors, nurses and other staff, especially with limited formal (undergraduate and postgraduate) training [4, 5].

An important starting point in effective clinical management is the assessment and identification of problems [6]. Unfortunately, practitioners often miss many of patients’ needs and symptoms, especially when these are non-physical [7–11]. This problem is compounded because patients have multiple symptoms and concerns [12–14]. Asking patients to report their concerns using Patient Reported Outcome Measures (PROMs) has been proposed as a way to overcome this [15]. When repeated over time, such assessments can become measurable outcomes, revealing how patients’ health status changed following the care provided [16].

Although practitioners express positive attitudes towards measuring PROMs [17, 18], routine measurement of concerns is hampered by a lack of training and guidance on how to use and respond to PROMs in clinical care [17–19]. Published guidance on using PROMs [20–22] is fragmented; it lacks specified steps and recommendations to follow when implementing PROMs [20, 21] or does not focus on advance disease [22]. Even more pressing, there is a lack of guidance on how to respond to specific PROM scores. There is often an unfamiliarity with the score interpretation [23], while PROM scores are potentially not fed back to the right person, or reported as often as needed [24]. Clinical significance of health-related quality of life scores are not always reported in studies [25, 26]. Unsurprisingly, therefore, clinical decision-making is not often based on outcome scores [27] and measuring PROMS seems to have a stronger impact on process-aspects of care, i.e. detection of symptoms, than on outcome-aspects of care, i.e. patients’ health status (e.g. [15, 23, 28, 29]). Ideally, PROMs should routinely be used and assist in detecting problems, planning treatment, and monitoring how well concerns are alleviated.

To fill this gap we need specific clinical decision support aids for specific PROM scores. This should be linked with better focused guidance on how to implement PROMs in routine clinical practice. It is important that both sets of guidance are developed together. This will ensure specific PROMs are successfully implemented in clinical care, while at the same time clinicians are supported in using the PROMs when responding to concerns.

One of the most widely used outcome measures in clinical practice in advanced illness is the Palliative (or Patient) care Outcome Scale (POS) family of measures (consisting of the POS, Integrated POS (IPOS), African Palliative Care Association African POS (APCA African POS), and POS-Symptoms (POS-S)) [30]. It assesses physical symptoms, emotional, psychological, and spiritual concerns, and needs for information and support [31]. It is brief (<10 minutes to complete), widely validated, able to transfer across settings, has good responsiveness to change, and has been translated and/or culturally adapted and revalidated in many different languages and cultures (e.g. [32–34]). A full suite of free user support resources is available at www.pos-pal.org [35]. During a training day on POS in 2013, we identified clinicians’ need for guidance in the interpretation of and responding to POS scores. Most difficulties were encountered with interpreting the questions regarding psychological functioning (i.e. depression/feeling worthwhile, n = 18/36 comments), information provision (n = 7/36 comments), and family anxiety (n = 4/36 comments). Breathlessness is a very disturbing symptom for patients [36, 37] and their families [38]. Creation of evidence-based clinical guidance on how to respond to these concerns is needed.

The aim of this study was therefore to develop a Clinical Decision Support Tool (CDST) for the specific POS items of most concern to clinicians: information needs, family anxiety, depression, and breathlessness. The guidance is aimed for all practitioners and settings, and is applicable to all patients/family caregivers with complex needs and progressive, life-threatening and serious disease. Because successful implementation of PROMs in clinical practice is a precondition to using a CDST, but current guidance is fragmented, in parallel we also developed accompanying guidance on PROM implementation. This article reports the development and final formats of the CDST and implementation guidance. It also offers a novel methodological approach for CDSTs in other areas.

## Methods

### Design

A systematic literature search and modified Delphi study were conducted to develop the CDST. A literature search and expert consultations were used to create the PROMs implementation guidance. Ethical approval was granted by the Research Ethical Committee of King’s College London (BDM/13/14-3). Participants were explained that participation implied informed consent.

### Clinical Decision Support Tool (CDST)

The CDST on how to respond to different levels of reported POS scores on information needs, family anxiety, depression, and breathlessness was created using a systematic literature search to develop preliminary recommendations for clinical care, followed by a modified Delphi approach to determine how the recommendations should be applied for different levels of POS score severity.

### Systematic literature search

#### Search strategy

To develop draft recommendations we searched for guidelines and systematic reviews on the aforementioned topics in PubMed, Google Scholar (first 4 pages), Cochrane Database, and the York DARE database (2000 to end June 2013). The websites of NICE (UK), National Guideline Clearinghouse (US), the Canadian Medical Association, and google.com (first 4 pages) were hand searched for relevant guidelines. Three guides on using PROMS were screened for relevant information [20–22] (see Additional file 1 for search strategies).

Inclusion criteria were:

i). Guidelines/systematic reviews focusing on general palliative care, or focusing on information needs, family anxiety, depression, or breathlessness in palliative care (sources focusing on palliative care in a specific setting were included)
ii). Guidelines/systematic reviews published in English/Dutch/German/Italian (languages available in the study team)
iii). Guidelines from national (disease) organizations
iv). Guidelines providing an evidence-base for created recommendation
v). Systematic reviews published in peer-reviewed journals

Exclusion criteria were guidelines/systematic reviews:

i). Not focusing solely on palliative care (but on the entire trajectory of disease(s))
ii). Focusing on a specific disease
iii). Focusing solely on pediatric care
iv). Of which an updated version was available
v). Of which no full-text was available

#### Data extraction

Data from included sources on how to clinically respond to information needs, family anxiety, depression, and breathlessness was extracted (by LV).

#### Analysis

LV drafted recommendations based on the extracted evidence. These were then critically revised by the co-authors (RH, CB, SP). Two authors (LV/IH) finalized the draft recommendations which were then taken forward into the subsequent modified Delphi study.

### Modified Delphi study

We conducted a two-round online Delphi-study [39, 40] (hosted via internet platform Keypoint), to appraise and revise the draft recommendations and agree an expert consensus on the appropriateness of all recommendations for all scores on the POS items.

#### Participants

Overall, 38 experts were purposefully sampled (we approached 48, of which 10 declined beforehand); comprising 26 clinicians, 24 researchers (some had a dual role), and 4 patient/family representatives (as experts by experience, recruited via the UK National Council of Palliative Care and the European Cancer Patient Coalition). Experts came from the UK, US, Netherlands, Italy, Germany, Australia, South Africa, Belgium, Greece, Sweden, Poland, and Switzerland. Experts were chosen based on their expertise in the field of the topics under study.

#### Ratings

In the Delphi round 1, experts rated the appropriateness of recommendations for all the different answer categories (0–4) for each POS-item and provided comments and/or suggested revisions. Appropriateness was rated on a 1–9 scale (‘not at all appropriate’ to ‘extremely appropriate’) with a ‘do not know’ option and space for comments. In round 2, a summary of the results of round 1 was provided (median, range, interquartile ranges, summary qualitative remarks) which participants were asked to take into account when rating the recommendations again. The results of round 1 were circulated. If recommendations in round 2 achieved a median ranging 7–9 and did not have >30 % of scores in the 1–3 and 7–9 range, they were deemed as appropriate [41–43] and included in the CDST. Lastly, the draft CDST was commented upon by (the same, plus one additional) experts via email and in a face-to-face research meeting (with different researchers).

#### Quality of evidence

For each recommendation, the quality of evidence was determined, using an adapted GRADE approach [44] with the classifications: A (e.g. meta-analysis, systematic review of randomized controlled trials (RCTs), RCT), B (e.g. cohort studies, case–control studies), C (e.g. retrospective, poor quality cohort studies), and D (qualitative studies, expert opinion) quality evidence. We rated each recommendation using the most recent and highest level of known evidence (based on design). If sources already provided a quality rating, their rating was used. Additional file 2 depicts how quality ratings from each source were mapped to the ABCD framework.

### Accompanying guidance on implementing PROMs

We scoped the literature for relevant guidance on implementing PROMs. Data was extracted from these sources to develop (with assistance of several experts in the field) a draft implementation guidance linked to our specific PROM, the POS. Next, we invited comments and critical revisions via email from the 10 members of the European Association for Palliative Care (EAPC) Taskforce Outcome Measures (we approached 13, of which three declined beforehand), plus one additional expert who agreed to participate, representing the countries of the UK, US, Germany, Australia, Italy, Belgium, and South Africa. A finalized version was created with comments from one additional expert.

## Results

### Clinical Decision Support Tool (CDST)

#### Systematic literature search

**Fig. 1:**
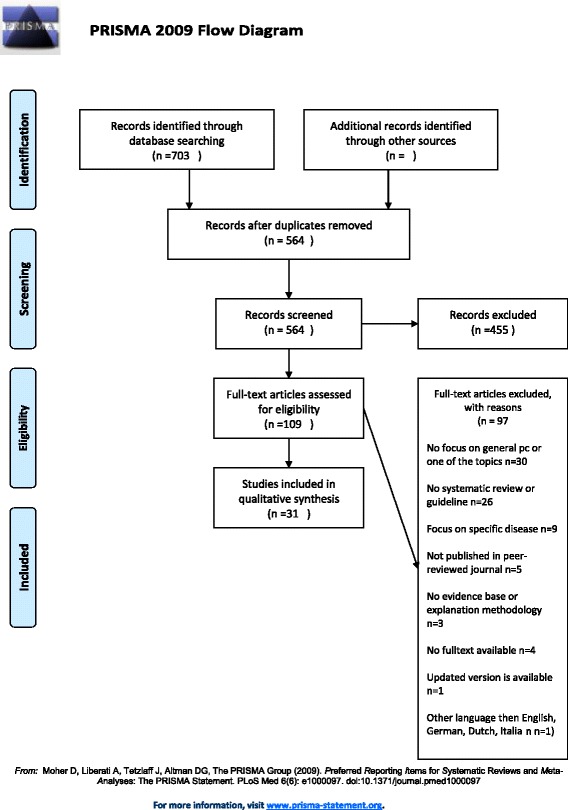
PRISMA flowchart inclusion guidelines/systematic reviews Clinical Decision Support Tool

Our search of the online databases revealed 703 sources (564 after duplication removal) of which 31 were included (see Fig. 1 for the flowchart of inclusion) [45–75]. Two additional guidelines [76, 77] were added as they were referred to by two included references [49, 73]. In addition, nine extra guidelines were included from the screened websites [78–86]. As panic/anxiety is often associated with breathlessness, we screened the NICE and Clearinghouse websites and all included guidelines for additional sources, revealing one additional source [87]. Therefore, ultimately 43 sources were included.

All included sources were scrutinized for evidence-based conclusions, data and recommendations on how to clinically respond to information needs, family anxiety, depression, and breathlessness in palliative care. Overall, 47 recommendations were drafted to be rated in the Delphi Round 1. Not all included sources provided information that could be used to draft recommendations, e.g. one review focusing on the effect of home-based palliative care found inconsistent results. As no other source focused on this topic, no recommendations about home-based care were made [72].

#### Modified Delphi study

In total, 25/38 (66 %) of our experts participated in round 1. Open comments were used to adjust wordings of several recommendations (services should be ‘offered’ instead of ‘provided’; pharmacological interventions should be ‘offered, alongside non-pharmacological interventions’). For round 2, experts were asked to take into account a summary of the qualitative and quantitative results of round 1. In this second round, 62 % (23/37, one person withdrew from the project) of the experts participated. Demographic characteristics of participating experts in rounds 1 and 2 are displayed in Additional file 3. Open comments (services should be provided, ‘depending on resources’; psycho-education, ‘i.e. teaching, explanation’) were used to finalize the recommendations, while the predefined cut-offs were used to draft the CDST. Quality of evidence was added for each recommendation, ranging from high to very low (later adapted to A–D). One recommendation (regarding chest-wall vibration to treat breathlessness) was downgraded from A to B as evidence was based on laboratory (as opposed to clinical) studies.

#### Final clinical decision support tool

**Fig. 2:**
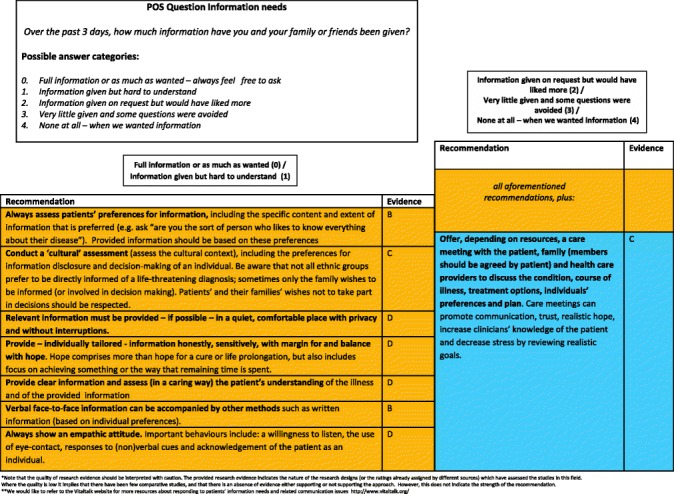
Final decision diagrams. Legends: POS score decision diagrams format 1. Information needs

**Fig. 3:**
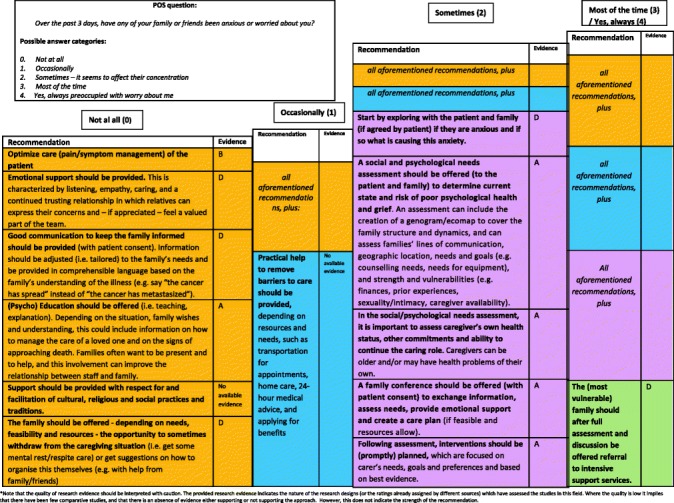
Final decision diagrams. Legends: POS score decision diagrams format 1. Family anxiety

**Fig. 4:**
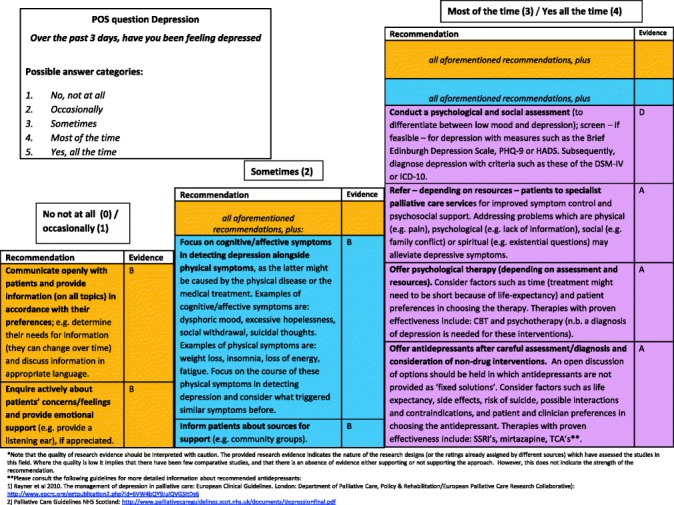
Final decision diagrams. Legends: POS score decision diagrams format 1. Depression

**Fig. 5:**
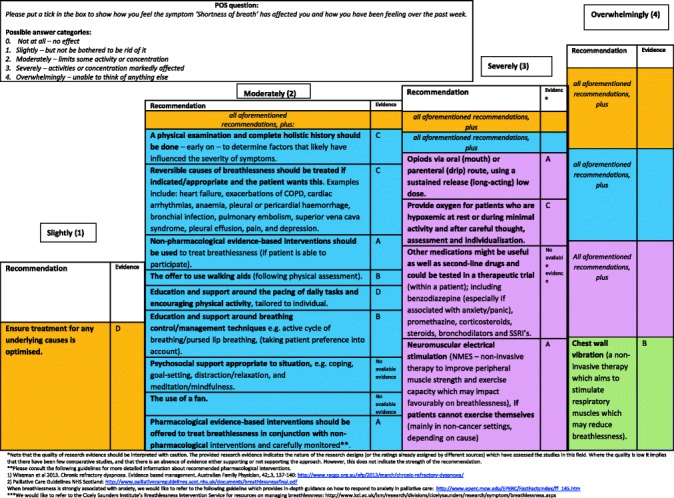
Final decision diagrams. Legends: POS score decision diagrams format 1. Breathlessness

**Fig. 6:**
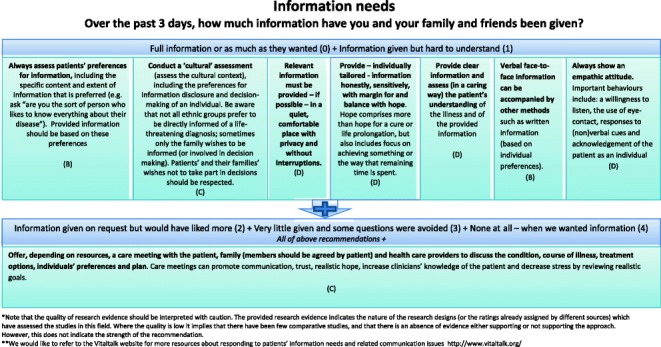
Final decision diagrams. Legends: POS score decision diagrams format 2. Information needs

**Fig. 7:**
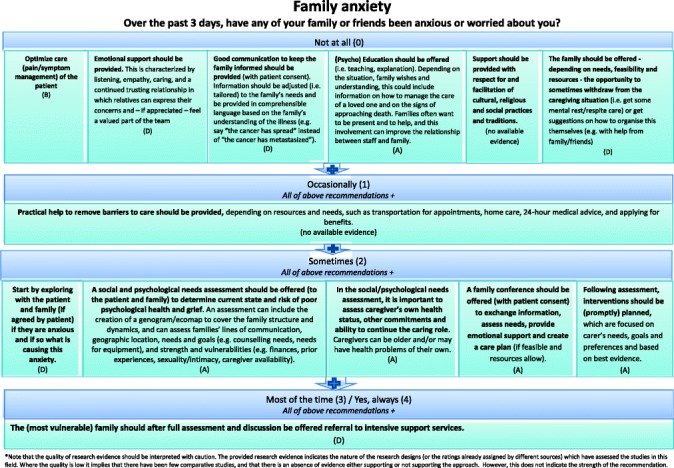
Final decision diagrams. Legends: POS score decision diagrams format 2. Family anxiety

**Fig. 8:**
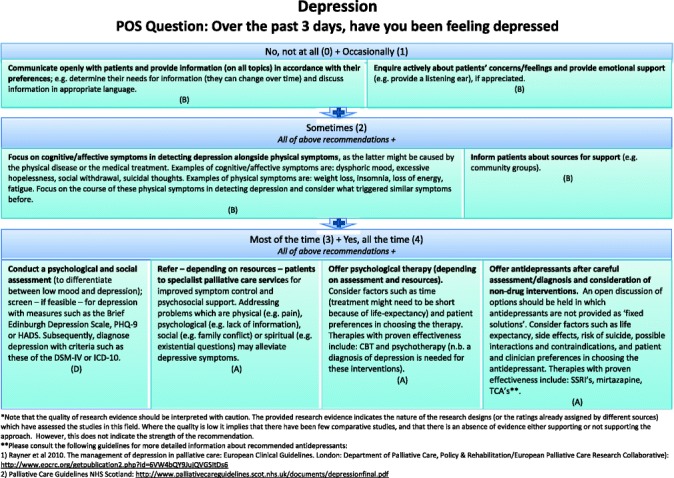
Final decision diagrams. Legends: POS score decision diagrams format 2. Depression

**Fig. 9:**
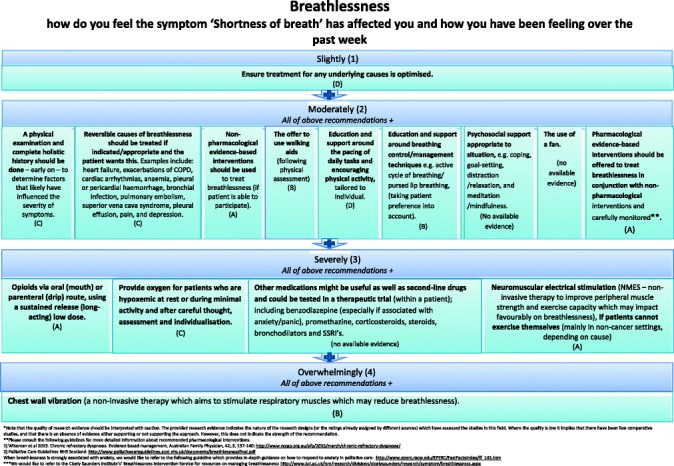
Final decision diagrams. Legends: POS score decision diagrams format 2. Breathlessness

Two formats of a decision-diagram for each POS item and a manual of the CDST were created and again sent out to all experts, of which 41 % (15/37) provided comments, in addition to one other expert in the field. Main comments focused on making it clear that for higher POS scores, lower recommendations still apply and creating both a short and long manual, while both formats of the decision diagrams were equally preferred (also in the face-to-face researchers meeting). The final decision-diagrams are depicted in Figs. 2, 3, 4, 5, 6, 7, 8 and 9. Additional file 4 depicts the short manual while the long manual can be found at the Palliative care Outcome Scale website [35]. Independent of POS score, core recommendations center on: i) good patient care, ii) the provision of psychosocial support and empathy, and iii) the use of open communication. Proper assessment is needed for increasing scores, followed by non-pharmacological interventions, and pharmacological interventions for high levels of depression and breathlessness.

### Implementation guidance

Our scoping identified four major guides which had complementary approaches: i) the PROMs guidelines of the International Society for Quality of Life Research (ISOQOL) including an 8-step framework [22], ii) the Outcome Measures in Palliative Care booklet [20], iii) the White paper on Outcome Measures of the EAPC [21], and iv) for the POS family of measures, the Guidelines for using the POS [88]. To provide guidance on implementing PROMs in clinical practice, the ISOQOL’ [22] 8-step framework was followed. The 8 steps include i) identify goals for collecting PROMs; ii) select patients, setting, and timing of assessment; iii) determine which questionnaire to use; iv) choose a mode for administering/scoring the questionnaire; v) design processes for reporting results, vi) identify aids to facilitate score interpretation; vii) develop strategies for responding to identified issues; and 8) evaluate the impact of measuring PROMs on practice. We amalgamated consistent recommendations from the different sources into this framework.

In total, 9/11 (82 %) of the invited participants provided comments on the guidance (in addition to one other expert in the field). The comments stressed the need to define the target group, present tables/boxes, and introductory passages, and these were integrated into the final guidance. The key recommendations of the implementation guidance are summarized in Table 1 for each step (the complete guidance can be found at [35]).

**Table 1:** Implementation guidance key recommendations

	Step	Key recommendations
1	Identify the goals for collecting Patient Reported Outcome Measures (PROMs)	Measuring PROMs can serve goals on different levels (several are often combined):
		Patient level goals: screening for symptoms and problems, monitoring of symptoms, aid decision making, facilitate communication with patients and within the team
		Service/setting level goals: evaluate and improve the quality of care (e.g. services), demonstrate effect, promote good practice
		Policy level goals: improving and monitoring palliative care practice on policy level (e.g. recommended routine collection and minimum dataset)
		POS measures
		Palliative care Outcome Scale (POS) measures can serve all discussed goals
2	Select patients, setting, and timing of assessment	Respondents: The ideal way to collect PROM data is patient report. In palliative care, this can be difficult, in which case proxy rating is used (family or professional). Measuring both patient and proxy ratings is ideal. Family carers’ own needs should be measured
		Setting: Measurement can be done both within/outside the clinical setting and within/between visits
		Timing: For screening PROMs are used once, for monitoring more often. Measurement frequency and questionnaire length should be related. Some argue that, ideally, no change in ‘window of measurement’ should be made. However, flexibility might be needed, e.g. following a change in situation or depending on patient preference
		POS measures
		POS measures have patient (all), family (POS), and staff (POS, IPOS) versions
		Both screening and monitoring is possible
		The measurement window of POS measures are either 3 ((APCA African) POS, IPOS) or 7 days (POS-S, IPOS). In practice, POS measures can be measured more flexibly, in response to clinical circumstances
3	Determine which questionnaire to use	Take several factors into consideration in choosing outcome measure e.g., aim of use, questionnaire available
		Choose outcome measure based on evidence, with sound psychometric properties and suited for the clinical task
		Use multidimensional (specific or generic) measures which allow for comparisons across settings and countries
		POS measures:
		POS (individual items and total score) has good psychometric properties. POS-S/APCA African POS are validated, IPOS is being validated
		POS measures are holistic, translated, can be used in various settings and diseases and in clinical practice (e.g. to enhance patient management and as a quality improvement tool)
4	Choose a mode for administering/scoring the questionnaire	PROMS can be collected using self- and interview-administration, while computer-completion is efficient
		Explain to patients why PROMS are helpful
		Pilot the measure with a few patients
		POS measures
		There are several ways to administer POS measures: i) leave the measure with the patient (provide written or verbal information), ii) stay with the patient (patient self-completes or practitioner helps), or iii) integrate measurement into holistic assessment (staff version – for specialists only)
5	Design processes for reporting results	PROM results should be shared with other health care practitioners (who can provide assistance in how to respond to certain issues) and the patient (it can integrate them as active member of the team)
		Decide how to present results, e.g. numerical info (easy to generate) and/or graphic representations (easy to interpret over time, but might be more difficult to integrate into standard workflow). Looking at scores over time is also important
		Deal with (and anticipate on) missing data, which might be more prominent with long, paper/self-administrated PROMs and large sets. Avoiding missing data is difficult in advanced disease, but can be anticipated upon by quality control procedures (e.g. double checking). Recommendations have been developed for handling missing data (see MORECare Statement [109])
		Store data in accordance with legal requirements
		POS measures
		Scores related to individual items and summary score can be generated. Summary scores highlight overall severity of needs, individual scores show where specific problems lie
		When analyzing, check your data and note missing values
6	Identify aids to facilitate score interpretation	For measures responsive to change use (and determine) the minimum clinically important difference (MCID – distinguishes between clinically relevant and statistically significant changes
		If available, published cut-off scores can help with interpreting scores
		Guidelines or disease management pathways can be linked to PROM scores, but clear guidance is unavailable for many symptoms/topics. They are simple to understand, but do not provide information about clinical importance of scores for an individual
		POS measures
		The interpretation of scores is guided by clinical expertise and patient’s condition.
		The MCID for the POS is a one-point change
7	Develop strategies for responding to identified issues	PROM scores should go into clinical notes, shared with clinicians/patients, and used to improve care and influence decision-making; exploration with patients can increase understanding but might be time-consuming
		PROM scores might be integrated with other clinical data
		Develop a routine for how PROM scores are used in ward rounds, team meetings, other consultations
		POS measures
		A Clinical Decision Support Tool for POS items information needs, family anxiety, depression, and breathlessness is developed
8	Evaluate the impact of measuring PROMs on practice	Precondition for successful implementation: use change management principles, facilitation, and communication to help embed PROM measurement in clinical practice
		Take into account described facilitators/barriers during preparing, implementing, and evaluating PROM measurement in clinical care
		Evaluate the impact of the PROM implementation, e.g. set up quality improvement initiatives (audits/benchmarking), use different (quasi/experimental) designs, evaluate implementation process, relate to quality indicators
		POS measures
		Ensure that staff is positive and see the added value of using POS, use a supportive training program to ensure routine uptake, and feed results back to sustain staff commitment

## Discussion

This is the first study to develop a CDST for some of the complex problems faced in advanced disease, in particular information needs, family anxiety, depression, and breathlessness, linking these to specific actions. The CDST has an evidence-based approach, responding to different levels of patient-reported symptoms. We found it was necessary to have both core recommendations, applicable for all symptom levels, as well as responses that could be titrated up with increasingly severe symptom scores. Accompanying guidance on PROM implementation was also developed, using an 8-step approach. By producing both types of evidence, PROMs (and specifically POS, or similar tools) might be more widely integrated in clinical care and their effects on patients’ outcomes can be strengthened.

The potential of our guidance and CDST to improve provided care and patient outcomes is supported by accumulating evidence. The Australian Palliative Care Outcome Collaboration showed that implementing PROMs nationally in palliative care improved patients’ reported symptoms year after year [89, 90]. Expert consensus underlines the importance of coming to a routinely collected set of outcome measures, which can be used to make comparisons across services and countries [21]. Our provided guidance might assist in this development while overcoming some of the perceived barriers of implementing PROMs, most notably a lack of knowledge and education [6, 91].

The CDST can assist clinicians in responding to the psychosocial aspects of clinical care which they may feel less confident in delivering. Across the several symptoms included in our Delphi, for higher scores, more intensive clinical responses seem warranted. Ensuring the core recommendations (good patient care; providing psychosocial support and empathy; the use of open communication) are in place remains vital in these situations. These core recommendations reflect previous study findings, showing that patients expect their clinicians to show both technical competence [92] as well as seeing them as an individual person [93]. Empathy becomes increasingly important in progressing disease [94], while most medical complaints are related to communication deficits [95, 96], adding not only to patient but also caregiver distress [97]. Our CDST postulates that proper assessment should be followed by non-pharmacological interventions which can be progressed to pharmacological interventions in the more medical oriented symptoms of breathlessness and depression. However, also in these domains, there seems a preference for psychosocial interventions. Indeed, a recent RCT showed that a short-term service provided by palliative care, respiratory, physiotherapy, and occupational therapy improved breathless patients’ outcomes [98]. This service combined pharmacological review with a focus on non-pharmacological interventions such as pacing and relaxation.

Although originally developed for the POS family of measures, the core recommendations point towards the potential usefulness of our CDST for other PROMs covering similar domains (e.g. the Edmonton Symptom Assessment System [99], the McGill Quality of Life Questionnaire [100], or quality of life measures such as the EORTC-QLQC30 [101]). It is a wider problem that in advanced illness patients’ and caregivers’ psychosocial needs often remain unmet [102, 103]. A recent systematic review, including but not limited to POS, concluded that feeding back PROM data in palliative care influenced patients’ psychosocial outcomes the most [104]. Our CDST might strengthen this finding by generalizing its applicability beyond POS.

Although the CDST proposals are based on scientific evidence combined with expert opinion, this tool is not intended to be prescriptive. Instead, it aims to help practitioners think through the best decision towards difficult and complex encountered problems. Being too prescriptive can be counterproductive, especially because in advanced illness patients and families have complex and often quite individual needs, circumstances, and trajectories, which interact [1, 105]. Instead the CDST should be used as the name implies, as a support, to aid a wider range of aspects to be considered when making treatment choices, alongside skilled individual clinical assessment and knowledge, taking into account patients’ and families’ individual preferences, circumstances and available resources. In areas of uncertainty or conflict, specialist support or a second opinion should be obtained. The CDST should not be used as an endpoint (‘tick-box’ exercise) in itself but as a starting point to achieve high quality person-centered care and good clinical practice. As the implementation guide makes clear, training and ongoing support will be an essential component.

This study has limitations. Our sample size was relatively small, although it should be noted that the responses rates in the online Delphi (66 % vs 62 %) are in line with a previous similar study in this field [106]. Next, we asked participants in the Delphi study to review a wide area of topics, which might have been challenging, especially surrounding the specific topic of breathlessness. However, as palliative care focuses on physical, psychological, emotional, and spiritual issues, we anticipated most participants to have (some) knowledge in all domains and added a ‘do not know’ option in the Delphi. The quality rating should also be interpreted with caution as we used an adapted GRADE approach. Many sources used different rating systems and we followed sources’ own evidence-levels if already provided. No strength of recommendation (representing whether desirable effects of recommendations clearly outweigh (or not) undesirable effects [44]) was provided due to the heterogeneity of used quality ratings and the envisaged applicability of our recommendations in different settings and countries. Lower ratings do not necessarily imply that these recommendations are not important, as they were among the highest rated recommendations by our expert panel (e.g. the provision of emotional support and respect for cultural/religious traditions when handling family anxiety). These ratings also highlight the pressing need for more high-quality research studies to build the evidence-base of these and other key components of palliative care. For example, the use of a fan was labelled as ‘no evidence’ based on a Cochrane review [59]. However, this is still in the early stages of evaluation, with recent studies showing conflicting effects (e.g. [107, 108]). As fans are inexpensive, unlike to cause any harm and have low side effects, they can be worth trying. Another limitation entailed that we only included sources that focused on palliative care in general and not on specific diseases as we sought recommendations that were applicable across diseases. For some diseases, the evidence might propose alternative strategies; however, we would expect experts in those fields to be aware of this evidence to take it into consideration. Although we used a broad search strategy, relevant sources might have been missed. Furthermore, we refer in footnotes to the Cicely Saunders Institute’s Breathlessness Intervention Service and the Vitaltalk organization for more resources on breathlessness and information needs. These were not found via our search strategies (as they are very recent), but we believed them to be of importance to clinical care. Finally, due to time and resource constraints, only one author identified resources and extracted data of included sources. That being said, a major strength of this work includes the involvement of patients and families in the development of the CDST and the combination of evidence-based recommendations with clinician expertise. Future studies can use this study as a starting point in developing evidence-based CDST for advanced disease, and should now refine our proposed CDST, test its effect on patient and family outcomes, and develop CDST for other POS items.

## Conclusions

Our findings underline the importance of providing good patient care, psychosocial support and empathy, and communication in advanced disease for all patients and families, irrespective of POS scores, in the domains of information needs, family anxiety, depression, and breathlessness. For increasing scores, patients’ symptoms should be assessed and responded to with non-pharmacological interventions, followed by pharmacological interventions (for breathlessness and depression). We were able to develop these recommendations into a CDST. Our novel evidenced-based approach to develop a CDST offers a replicable method for other areas. By using the presented recommendations alongside skilled clinical knowledge and patient preferences and ongoing training, it aims to help support clinicians provide the best possible patient-centered care and patients and their families achieve the best possible outcomes in highly threatening times. Systematically following the 8-step framework can support successful implementation of PROMS, and POS, in advanced disease.

## Additional files

Additional file 1:
**Search strategy.** (DOCX 26 kb) Additional file 2:
**ABCD approach quality of evidence.** (DOCX 53 kb) Additional file 3:
**Demographics participants.** (DOCX 18 kb) Additional file 4:
**Short manual of Clinical Decision Support Tools.** (DOCX 4651 kb)

## Abbreviations

CDST: Clinical decision support tool
EAPC: European Association for Palliative Care
ISOQOL: International Society for Quality of Life Research
POS: Palliative (or patient) Outcome Scale
PROMs: Patient reported outcome measures
