# Supplementary material for: How should we manage information needs, family anxiety, depression, and breathlessness for those affected by advanced disease: development of a Clinical Decision Support Tool using a Delphi design
Source: BMC Med. 2015 Oct 13;13:263. doi: 10.1186/s12916-015-0449-6 (PMC4604738; doi:10.1186/s12916-015-0449-6)
Supplement: Additional file 1: — Search strategy. (DOCX 26 kb) [file 12916_2015_449_MOESM1_ESM.docx]

**Additional File 1 - Search strategy**

**Search strategy online databases (Pubmed, google scholar, Cochrane Database, York/Dare Association)**

| **Database** | **Search strategy** | **Filters** | **Date** |
| --- | --- | --- | --- |
| ***Imported into endnote:*** |  |  |  |
| ***Pubmed*** |  |  |  |
| *Information* | guideline AND palliative care AND information  ("guideline"[Publication Type] OR "guidelines as topic"[MeSH Terms] OR "guideline"[All Fields]) AND ("palliative care"[MeSH Terms] OR ("palliative"[All Fields] AND "care"[All Fields]) OR "palliative care"[All Fields]) AND information[All Fields] AND ("2000/01/01"[PDAT] : "3000/12/31"[PDAT]) | from 2000/01/01 | 25-6-2013 |
| *Information* | information needs AND palliative care  (information[All Fields] AND ("health services needs and demand"[MeSH Terms] OR ("health"[All Fields] AND "services"[All Fields] AND "needs"[All Fields] AND "demand"[All Fields]) OR "health services needs and demand"[All Fields] OR "needs"[All Fields])) AND ("palliative care"[MeSH Terms] OR ("palliative"[All Fields] AND "care"[All Fields]) OR "palliative care"[All Fields]) AND (systematic[sb] AND ("2000/01/01"[PDAT] : "3000/12/31"[PDAT])) | systematic reviews  from 2000/01/01 | 25-6-2013 |
| *Family anxiety* | guideline AND (family distress OR family anxiety) AND palliative care  ("guideline"[Publication Type] OR "guidelines as topic"[MeSH Terms] OR "guideline"[All Fields]) AND ((("family"[MeSH Terms] OR "family"[All Fields]) AND distress[All Fields]) OR (("family"[MeSH Terms] OR "family"[All Fields]) AND ("anxiety"[MeSH Terms] OR "anxiety"[All Fields]))) AND ("palliative care"[MeSH Terms] OR ("palliative"[All Fields] AND "care"[All Fields]) OR "palliative care"[All Fields]) AND ("2000/01/01"[PDAT] : "3000/12/31"[PDAT]) | from 2000/01/01 | 25-6-2013 |
| *Family anxiety* | palliative care AND (family distress OR family anxiety)  ("palliative care"[MeSH Terms] OR ("palliative"[All Fields] AND "care"[All Fields]) OR "palliative care"[All Fields]) AND ((("family"[MeSH Terms] OR "family"[All Fields]) AND distress[All Fields]) OR (("family"[MeSH Terms] OR "family"[All Fields]) AND ("anxiety"[MeSH Terms] OR "anxiety"[All Fields]))) AND (systematic[sb] AND ("2000/01/01"[PDAT] : "3000/12/31"[PDAT])) | from 2000/01/01  systematic reviews | 25-6-2013 |
| *Depression* | guideline AND depression AND palliative care  ("guideline"[Publication Type] OR "guidelines as topic"[MeSH Terms] OR "guideline"[All Fields]) AND ("depressive disorder"[MeSH Terms] OR ("depressive"[All Fields] AND "disorder"[All Fields]) OR "depressive disorder"[All Fields] OR "depression"[All Fields] OR "depression"[MeSH Terms]) AND ("palliative care"[MeSH Terms] OR ("palliative"[All Fields] AND "care"[All Fields]) OR "palliative care"[All Fields]) AND ("2000/01/01"[PDAT] : "3000/12/31"[PDAT]) | from 2000/01/01 | 25-6-2013 |
| *Depression* | depression AND palliative care  ("depressive disorder"[MeSH Terms] OR ("depressive"[All Fields] AND "disorder"[All Fields]) OR "depressive disorder"[All Fields] OR "depression"[All Fields] OR "depression"[MeSH Terms]) AND ("palliative care"[MeSH Terms] OR ("palliative"[All Fields] AND "care"[All Fields]) OR "palliative care"[All Fields]) AND (systematic[sb] AND ("2000/01/01"[PDAT] : "3000/12/31"[PDAT])) | from 2000/01/01  systematic reviews | 25-6-2013 |
| *Breathlessness* | guideline AND (breathlessness OR dyspnea) AND palliative care  ("guideline"[Publication Type] OR "guidelines as topic"[MeSH Terms] OR "guideline"[All Fields]) AND (("dyspnea"[MeSH Terms] OR "dyspnea"[All Fields] OR "breathlessness"[All Fields]) OR ("dyspnoea"[All Fields] OR "dyspnea"[MeSH Terms] OR "dyspnea"[All Fields])) AND ("palliative care"[MeSH Terms] OR ("palliative"[All Fields] AND "care"[All Fields]) OR "palliative care"[All Fields]) AND ("2000/01/01"[PDAT] : "3000/12/31"[PDAT]) | from 2000/01/01 | 25-6-2013 |
| *Breathlessness* | Palliative care AND (breathlessness OR dyspnea)  ("palliative care"[MeSH Terms] OR ("palliative"[All Fields] AND "care"[All Fields]) OR "palliative care"[All Fields]) AND (("dyspnea"[MeSH Terms] OR "dyspnea"[All Fields] OR "breathlessness"[All Fields]) OR ("dyspnoea"[All Fields] OR "dyspnea"[MeSH Terms] OR "dyspnea"[All Fields])) AND (systematic[sb] AND ("2000/01/01"[PDAT] : "3000/12/31"[PDAT])) | from 2000/01/01  systematic reviews | 25-6-2013 |
| ***Google scholar*** |  |  |  |
| *Information* | guidelines palliative care information  http://scholar.google.co.uk/scholar?as_q=guidelines+palliative+care+information&as_epq=&as_oq=&as_eq=&as_occt=any&as_sauthors=&as_publication=&as_ylo=2000&as_yhi=&btnG=&hl=en&as_sdt=0%2C5 | From 2000  First 4 pages were searched | 25-6-2013 |
| *Family anxiety* | guidelines palliative care family distress family anxiety  http://scholar.google.co.uk/scholar?q=guidelines+palliative+care+family+distress+family+anxiety&btnG=&hl=en&as_sdt=0%2C5&as_ylo=2000 | From 2000  First 4 pages were searched | 25-6-2013 |
| *Depression* | guidelines palliative care depression  http://scholar.google.co.uk/scholar?q=guidelines+palliative+care+depression&btnG=&hl=en&as_sdt=0%2C5&as_ylo=2000 | From 2000  First 4 pages were searched | 25-6-2013 |
| *Breathlessness* | guidelines palliative care breathlessness dyspnea  http://scholar.google.co.uk/scholar?q=guidelines+palliative+care+breathlessness+dyspnea+&btnG=&hl=en&as_sdt=0%2C5&as_ylo=2000 | From 2000  First 4 pages were searched | 25-6-2013 |
| ***Cochrane Database*** | <http://www.thecochranelibrary.com/view/0/index.html>  search term ‘palliative’ | No data restriction, is not possible (so 2 hits from prior 2000) | 25-6-2013 |
| ***York/DARE association*** |  |  |  |
| *Information* | guideline AND palliative care AND information | 2000 to 2013 | 26-6-2013 |
| *Information* | information needs AND palliative care | 2000 to 2013 | 26-6-2013 |
| *Family anxiety* | guideline AND (family distress OR family anxiety) AND palliative care  (in all fields) | 2000 to 2013 | 26-6-2013 |
| *Family anxiety* | palliative care AND (family distress OR family anxiety) | 2000 to 2013 | 26-6-2013 |
| *Depression* | guideline AND depression AND palliative care | 2000 to 2013 | 26-6-2013 |
| *Depression* | depression AND palliative care  ((depression) AND (palliative care)) and ((Systematic review:ZDT and Bibliographic:ZPS) OR (Systematic review:ZDT and Abstract:ZPS) OR (Cochrane review:ZDT) OR (Cochrane related review record:ZDT)) FROM 2000 TO 2013 | 2000 to 2013  CDR assessed review (bibliographic)  CRD assessed review (full abstract)  Cochrane review  Cochrane related review record | 26-6-2013 |
| *Breathlessness* | guideline AND (breathlessness OR dyspnea) AND palliative care | 2000 to 2013 | 26-6-2013 |
| *Breathlessness* | Palliative care AND (breathlessness OR dyspnea)  ((palliative care) AND (breathlessness OR dyspnea)) and ((Systematic review:ZDT and Bibliographic:ZPS) OR (Systematic review:ZDT and Abstract:ZPS) OR (Cochrane review:ZDT) OR (Cochrane related review record:ZDT)) FROM 2000 TO 2013 | 2000 to 2013  CDR assessed review (bibliographic)  CRD assessed review (full abstract)  Cochrane review  Cochrane related review record | 26-6-2013 |

**Search strategy websites**

| **Source** | **Website + how browsed.** | **Date** |
| --- | --- | --- |
| ***Not imported into endnote*** |  |  |
| ***NICE*** | nice.org.uk  <http://www.nice.org.uk/guidance/index.jsp?action=byType>  All the guidelines under all types of guidance were browsed  No restriction for date possible | 25-6-2013 |
| ***National Guideline Clearinghouse*** | [www.guideline.gov](http://www.guideline.gov)  Guidelines – search term ‘palliative’  <http://guideline.gov/search/search.aspx?term=palliative>  No restriction for date possible | 25-6-2013 |
| ***Canadian Medical Association*** | <http://www.cma.ca/index.php>  Search:  Keyword ‘palliative’  Restriction date: as of 1-1-2000 | 25-6-2013 |
| ***Google*** | Google.go.uk guideline palliative care (<https://www.google.co.uk/#q=guideline+palliative+care&ei=7nbVUZHxIoyT0AW0xICoAw&start=20&sa=N&bav=on.2,or.r_qf.&fp=b76b2d48273e04ee&biw=1280&bih=930>)  First 4 pages were searched. | 26-6-2013 |

**Extra guidelines regarding anxiety/panic**

As breathlessness is often associated with anxiety, we decided to include a link to anxiety/panic guidelines in the part of the DST focussing on breathlessness. The websites of NICE and Clearinghouse were searched using the following approach:

| **Source** | **Website + how browsed.** | **Date** |
| --- | --- | --- |
| ***NICE*** | nice.org.uk  search for “anxiety panic”  Information type: “guidance”  Results: 31 hits | 15-10-2013 |
| ***National Guideline Clearinghouse*** | <http://www.guideline.gov/search/search.aspx?term=panic+anxiety>  guideline.gov  than search for: “panic anxiety”  Results: 31 hits | 15-10-2013 |

Moreover, we searched all the guidelines we included to see whether they provided any references to guidelines on panic/anxiety.
