# Supplementary material for: How should we manage information needs, family anxiety, depression, and breathlessness for those affected by advanced disease: development of a Clinical Decision Support Tool using a Delphi design
Source: BMC Med. 2015 Oct 13;13:263. doi: 10.1186/s12916-015-0449-6 (PMC4604738; doi:10.1186/s12916-015-0449-6)
Supplement: Additional file 2: — ABCD approach quality of evidence. (DOCX 53 kb) [file 12916_2015_449_MOESM2_ESM.docx]

**Additional File 2 – Adopted ABCD Framework**

**Original GRADE quality of evidence and definitions**

| A | High quality | further research is very unlikely to change our confidence in the estimate of effect |
| --- | --- | --- |
| B | Moderate quality | further research is likely to have an important impact on our confidence in the estimate of effect and may change the estimate |
| C | Low quality | further research is very likely to have an important impact on our confidence in the estimate of effect and is likely to change the estimate |
| D | Very low quality | any estimate of effect is very uncertain |

*(adapted from: p 926, Guyatt et al. GRADE: an emerging consensus on rating quality of evidence and strength of recommendations. BMJ 336:924-926 2008)(*[*1*](#_ENREF_1)*)*

**Adaptation GRADE for included sources**

| **Included source** | **Used quality of evidence** | **Adaptation quality of evidence to GRADE/ABCD framework** |
| --- | --- | --- |
| Guidelines for a Palliative Approach in Residential Aged Care, Australian Government, national health and medical Research Council 2006([2](#_ENREF_2))  *(based on Currow DC, Hegarty M: Residential aged-care facility palliative care guidelines: improving care. Int J Palliat Nurs 12:231-3, 2006)(*[*3*](#_ENREF_3)*)* | Levels: I, II, III-1, III-2, III-3, IV based on NHMRC for quantitative studies.  For qualitative evidence Level QE and for expert opinion | I – a systematic review of all relevant RCTs  II – at least one properly designed RCT  III-1 – well-designed pseudo-RCTs  III-2 – comparative studies with concurrent controls and allocation not randomised, case-control studies, or interrupted time series with a control group  III-3 – Comparative studies with historical control, two or more single-arm studies, or interrupted time series without a parallel control group  IV – case series, either post-test or pre-test and post-test  QE – qualitative evidence  EO – expert opinion (not used as basis) |
| Working group for the Clinical Practice Guidelines of Palliative Care. Clinical Practice Guideline for Palliative Care. Full version. Madrid: National Plan for the NHS of the MSC. Health Technologies Assessment Agency of the Basque Country, 2008. Clinical practice guidelines in the Spanish NHS. Osteba. No 2006/08([4](#_ENREF_4)) | ABCD | A – at least one meta-analysis, systematic review or clinical trial rated as 1 ++ (i.e. high quality meta-analysis, systematic review of clinical trials, or clinical trials with a very low risk of bias)and directly applicable to the target population or the guideline; or a body of evidence consisting of studies rated as 1 + (i.e. well-conducted meta-analysis, systematic review of clinical trials or well-conducted clinical trials with a low risk of bias) and demonstrating overall consistency of results  B – A body of scientific evidence including studies rated as 2++ (i.e. high quality systematic reviews of case control or cohort or studies. High quality case control or cohort studies with a very low risk of bias and a high probability that the relationship is causal), directly applicable to the target population of the guideline, and demonstrating overall consistently of results. Or extrapolated evidence from studies rated as 1++ (i.e. high quality meta-analysis, sr of clinical trials or clinical trials) or 1+ (well-conducted meta-analysis, sr of clinical trials or clinical trials)  C –Body of scientific evidence including studies rated as 2+ (well-conducted case-control or cohort studies with a low risk of bias and moderate probability that relationship is causal), directly applicable to target population of the guideline and demonstrating overall consistency of results; or extrapolated scientific evidence from studies rated as 2++ (high quality sr of case control/cohort tudies. High quality case control/cohort studies)  D – scientific evidence level 3 / 4 (non-analytic studies such as case reports and case series), or extrapolated  evidence from studies rated as 2+ (well-conducted case control/cohort)  BCP = Best clinical practice (recommended practice based on the clinical experience of the guideline development group) |
| Rayner L, Higginson I, Price A, et al: The management of depression in palliative care: European clinical guidelines. Department of Palliative Care, Policy & Rehabilitation, London 2010 ([5](#_ENREF_5)) | GRADE approach | Grade of evidence (high, moderate, low, very low) and strength of recommendation (weak, strong) |
| Palliative care guidelines: Depression. Depression in Palliative Care. NHS Lothian. Re-issue date: August 2010. Review date: August 2013 ([6](#_ENREF_6))  Palliative care guidelines: Breathlessness. Breathlessness in Palliative Care. NHS Lothian. Re-issue date: August 2010. Review date: August 2013 ([7](#_ENREF_7))  Palliative Care Guidelines. Pocket Edition 2010. Third edition/Version 2. NHS Lothian. ([8](#_ENREF_8)) | no strength of recommendation |  |
| Palliative care guidelines. Breaking bad news. ([9](#_ENREF_9)) <http://book.pallcare.info/index.php?tid=109>  Palliative care guidelines. Depression ([10](#_ENREF_10))  <http://book.pallcare.info/index.php?tid=49>  Palliative care guidelines. Dyspnoea([11](#_ENREF_11))  <http://book.pallcare.info/index.php?tid=41> | no strength of recommendation |  |
| National Consensus Project for Quality Palliative Care. Clinical practice guidelines for quality palliative care. Third Edition. Pittsburgh (PA),2013([12](#_ENREF_12)) | no strength of recommendation |  |
| Institute for Clinical Systems Improvement (ICSI). Palliative care. Bloomington (MN): Institute for Clinical Systems Improvement (ICSI); 2011 Nov([13](#_ENREF_13)) | GRADE approach | Quality of evidence (high, medium, low) + strength of recommendation (weak, strong)  *(very low is not included)* |
| Qaseem A, Snow V, Shekelle P, et al: Evidence-based interventions to improve the palliative care of pain, dyspnea, and depression at the end of life: a clinical practice guideline from the American College of Physicians. Ann Intern Med 148:141-6, 2008([14](#_ENREF_14)) | GRADE approach | High, moderate low (no very low) and Insufficient . (weak strong) |
| Wiseman R, Rowett D, Allcroft P, et al: Chronic refractory dyspnoea: Evidence based management. Australian Family Physician 42:137, 2013([15](#_ENREF_15)) | NHMRC (National Health and Medical Research Council) approach | Level 1 – meta-analysis  Level 4 – case series  *(level 2 and 3 are not included)* |
| Bausewein C, Booth S, Gysels M, et al: Non-pharmacological interventions for breathlessness in advanced stages of malignant and non-malignant diseases. Cochrane Database Syst 2008 ([16](#_ENREF_16)) | Adaptation of scale (Ebel 2004, Gomes 2006) | High strength = min 3 high quality studies which had performed multivariate analysis and >70% of studies had reported similar findings  Moderate = min 3 high quality studies reported <70% similar findings or a minimum of 3 medium quality studies and >50% reported similar findings  Low evidence = all other studies |
| Parker SM, Clayton JM, Hancock K, et al: A systematic review of prognostic/end-of-life communication with adults in the advanced stages of a life-limiting illness: patient/caregiver preferences for the content, style, and timing of information. J Pain Symptom Manage 34:81-93, 2007([17](#_ENREF_17)) | NHMRC (National Health and Medical Research Council) | IV a – evidence obtained from descriptive studies of provider practices, patient behaviours, knowledge, attitudes or a systematic review of the descriptive studies. |
| Hudson P, Remedios C, Zordan R, et al: Clinical Practice Guidelines for the Psychosocial and Bereavement Support of Family Caregivers of Palliative Care Patients. Melbourne, Australia, Centre for Palliative Care, St. Vincent's Hospital Melourne, 2010 ([18](#_ENREF_18)) *(based on: Hudson P, Remedios C, Zordan R, et al: Guidelines for the psychosocial and bereavement support of family caregivers of palliative care patients. J Palliat Med 15:696-702, 2012)(*[*19*](#_ENREF_19)*)* | NHMRC grades + 2 extra grades for qualitative studies/expert opinion | A – body of evidence can be trusted to guide practice  B – body of evidence can be trusted to guide practice in most situations  C – body of evidence provides some support for recommendation(s) but care should be taken with its application  D – body of evidence is weak and recommendation(s) must be applied with caution  E – Qualitative research (not NHMRC grade)  F – expert opinion (not an NHMRC grade) incl books, theoretical papers, non-systematic reviews and guidelines |
| Scheunemann LP, McDevitt M, Carson SS, et al: Randomized, controlled trials of interventions to improve communication in intensive care: a systematic review. Chest 139:543-54, 2011([20](#_ENREF_20)) | No strength of evidence |  |
| Davidson JE, Powers K, Hedayat KM, et al: Clinical practice guidelines for support of the family in the patient-centered intensive care unit: American College of Critical Care Medicine Task Force 2004-2005. Critical care medicine 35:605-622, 2007([21](#_ENREF_21)) | Cochrane approach  ABCD = grade of recommendation  1abc,2abc,3ab,4,5=level of evidence | A (1a) = systematic review of RCT  A(1b)=individual RCT  A (1c)=when all patients died before treatment, now some survive or when some survived, now all  B (2a)=SR of cohort studies  B (2b)=invidual cohort study  B (2c)=outcomes research  B (3a)=SR of case-control studies  B (3b)=individual case-control study  C (4)=case-series (and poor quality cohort and case-control)  D (5) = expert opinion without explicit critical appraisal, or base on physiology, bench research or fist principles.  *Please note: more elaborate information can be find in the article of Davidson et al., (Table 1), this is a summary of the most important points* |
| Candy B, Jones L, Drake R, et al: Interventions for supporting informal caregivers of patients in the terminal phase of a disease. Cochrane Database Syst Rev:CD007617, 201([22](#_ENREF_22)) | No strength of evidence |  |
| Plonk WM, Jr., Arnold RM: Terminal care: the last weeks of life. J Palliat Med 8:1042-54, 2005([23](#_ENREF_23)) | Use of SORT grades (Ebell et al 2004)  Not all results are scored, only the most important recommendations. | A-level = recommendation based on consistent and good quality  patient-oriented evidence (SR/meta-analysis of high quality studys + high quality cohort study, SR/meta-analysis of RCT, RCT, SR/meta-analysis of good quality cohort study, prospective cohort with good follow up);  B-level = Recommendation based on inconsistent or limited quality patient-oriented evidence (patient-oriented included outcomes that matter to patients, morbidity, mortaility, symptom improvement, cost reduction, quality of life). (SR/meta-analysis of lower quality studies or study with inconsistent findings, lower quality cohort or case-control, SR/meta analy sis of lower quality clinical trials or of studies with inconsistent findings, lower quality clinical trial, cohort study, case-control study), SR/meta-analysis of lower quality cohort study or with inconsistent results, retrospective cohort study or prospective with poor follow up, case-control, case-series)  C-level = Recommendation based on consensus, usual practice, opinion, disease-oriented evidence, case series for studies of diagnosis, treatment, prevention or screening |
| Andershed B: Relatives in end-of-life care--part 1: a systematic review of the literature the five last years, January 1999-February 2004. J Clin Nurs 15:1158-69([24](#_ENREF_24)) | No strength of evidence |  |
| Ostlund U, Brown H, Johnston B: Dignity conserving care at end-of-life: a narrative review. Eur J Oncol Nurs 16:353-67, 2012([25](#_ENREF_25)) |  | Evidence mainly consist of studies using qualitative methodology *(so that would be low)* |
| Hudson PL, Remedios C, Thomas K: A systematic review of psychosocial interventions for family carers of palliative care patients. BMC Palliat Care 9:17, 2010([26](#_ENREF_26)) |  | RCTs or review of RCTs (strong evidence)  1A. calculation of sample size and accurate SD of appropriate outcome variables  1B accurate and standard definition of appropriate outcome variables.  1C. Neither of above  Prospective study with comparison group (Non randomised trial, good observational, retrospectiy stuy controlling for confounding (fairly strong evidence)   1. Calculation of sample size and accurate standard definition of appropriate outcome variables and adjustment for the effect of important confounding variables 2. One or more of the above   Retrospective or observational studies (weaker evidence)   1. Comparison group, calculation of sample size, accurate and sd of appropriate outcome variables. 2. Two or more of the above 3. None of these   Cross-sectional study, Delphi exercise, consensus of experts (weak evidence) |
| Jennings AL, Davies AN, Higgins JP, et al: Opioids for the palliation of breathlessness in terminal illness. Cochrane Database Syst Rec 2001([27](#_ENREF_27)) | Cochrane | Randomised, double-blind, controlled trials were included |
| Mahler DA, Selecky PA, Harrod CG, et al: American College of Chest Physicians consensus statement on the management of dyspnea in patients with advanced lung or heart disease. Chest 137:674-91, 2010([28](#_ENREF_28)) | Delphi | Delphi (based on systematic review) |
| Gallagher R, Roberts D: A systematic review of oxygen and airflow effect on relief of dyspnea at rest in patients with advanced disease of any cause. J Pain Palliat Care Pharmacother 18:3-15([29](#_ENREF_29)) |  | Review of grade 4 studies (case series (and poor quality cohort and case-control studies)) |
| Booth S, Wade R, Johnson M, et al: The use of oxygen in the palliation of breathlessness. A report of the expert working group of the Scientific Committee of the Association of Palliative Medicine. Respir Med 98:66-77, 2004([30](#_ENREF_30)) |  | Delphi + Review |
| Simon ST, Higginson IJ, Booth S, et al: Benzodiazepines for the relief of breathlessness in advanced malignant and non-malignant diseases in adults. Cochrane Database Syst Rev:CD007354([31](#_ENREF_31)) | Cochrane | RTC and controlled clinical trials |
| Ujeyl M, Muller-Oerlinghausen B: [Antidepressants for treatment of depression in palliative patients : a systematic literature review]. Schmerz 26:523-36, 2012([32](#_ENREF_32)) |  | SR of RCTs |

*Please note that when source already provided information about the quality of evidence themselves, we followed their quality rating instead of the design rating. For example, there were many (non)pharmacological recommendations for how to treat breathlessness, which had a moderate evidence but were derived from a high design. In this example a moderate quality rating would be attributed.*

1. Guyatt GH, Oxman AD, Vist GE, Kunz R, Falck-Ytter Y, Alonso-Coello P, et al. GRADE: an emerging consensus on rating quality of evidence and strength of recommendations. BMJ 2008; 336: 924-6.

2. Guidelines for a Palliative Approach in Residential Aged Care, Australian Government, national health and medical Research Council, 2006

3. Currow DC, Hegarty M. Residential aged-care facility palliative care guidelines: improving care. Int J Palliat Nurs 2006; 12: 231-3

4. Working group for the Clinical Practice Guidelines of Palliative Care. Clinical Practice Guideline for Palliative Care. Full version. Madrid: National Plan for the NHS of the MSC. Health Technologies Assessment Agency of the Basque Country, 2008. Clinical practice guidelines in the Spanish NHS. Osteba. No 2006/08

5. Rayner L, Higginson I, Price A, Hotopf M. The management of depression in palliative care: European clinical guidelines. London: Department of Palliative Care, Policy & Rehabilitation, London / European Palliative Care Research Collaborative; 2010

6. Palliative care guidelines: Depression. Depression in Palliative Care. NHS Lothian. Re-issue date: August 2010. Review date: August 2013

7. Palliative care guidelines: Breathlessness. Breathlessness in Palliative Care. NHS Lothian. Re-issue date: August 2010. Review date: August 2013

8. Palliative Care Guidelines. Pocket Edition 2010. Third edition/Version 2. NHS Lothian. Re-issue date: August 2010. Review date: August 2013 2010

9. Palliative care guidelines. Breaking bad news. Palliative care adult network guidelines http://book.pallcare.info/index.php?tid=109 (Accessed on 25-03-2015)

10. Palliative care guidelines. Depression. Palliative care adult network guidelines http://book.pallcare.info/index.php?tid=49 (Accessed on 25-03-2015)

11. Palliative care guidelines. Dyspnoea. Palliative care adult network guidelines http://book.pallcare.info/index.php?tid=41 (Accessed on 25-03-2015)

12. National Consensus Project for Quality Palliative Care. Clinical practice guidelines for quality palliative care. Third Edition. Pittsburgh (PA),2013

13. McCusker et al. Institute for Clinical Systems Improvement (ICSI). Health care guideline: Palliative Care. Bloomington (MN). Fourth Edition November 2011

14. Qaseem A, Snow V, Shekelle P, Casey DE, Jr., Cross JT, Jr., Owens DK, Dallas P, Dolan NC, Forciea MA, et al. Evidence-based interventions to improve the palliative care of pain, dyspnea, and depression at the end of life: a clinical practice guideline from the American College of Physicians. Ann Intern Med 2008; 148: 141-6

15. Wiseman R, Rowett D, Allcroft P, Abernethy A, Currow D. Chronic refractory dyspnoea: Evidence based management. Aust Fam Physician 2013; 42: 137-40

16. Bausewein C, Booth S, Gysels M, Higginson I. Non-pharmacological interventions for breathlessness in advanced stages of malignant and non-malignant diseases. Cochrane Database Syst Rev 2008: CD005623.

17. Parker SM, Clayton JM, Hancock K, Walder S, Butow PN, Carrick S, Currow D, Ghersi D, Glare P, Hagerty R, Tattersall MH. A systematic review of prognostic/end-of-life communication with adults in the advanced stages of a life-limiting illness: patient/caregiver preferences for the content, style, and timing of information. J Pain Symptom Manage 2007; 34: 81-93

18. Hudson P, Remedios C, Zordan R, Thomas K, Clifton K, Crewdson M, Hall C, Trauer T, Bolleter A, Clarke D. Clinical Practice Guidelines for the Psychosocial and Bereavement Support of Family Caregivers of Palliative Care Patients. Melbourne, Australia: Centre for Palliative Care, St. Vincent's Hospital Melbourne, 2010

19. Hudson P, Remedios C, Zordan R, Thomas K, Clifton D, Crewdson M, Hall C, Trauer T, Bolleter A, Clarke DM, Bauld C. Guidelines for the psychosocial and bereavement support of family caregivers of palliative care patients. J Palliat Med 2012; 15: 696-702

20. Scheunemann LP, McDevitt M, Carson SS, Hanson LC. Randomized, controlled trials of interventions to improve communication in intensive care: a systematic review. Chest 2011; 139: 543-54

21. D Davidson JE, Powers K, Hedayat KM, Tieszen M, Kon AA, Shepard E, Spuhler V, Todres ID, Levy M, Barr J. Clinical practice guidelines for support of the family in the patient-centered intensive care unit: American College of Critical Care Medicine Task Force 2004-2005. Crit Care Med 2007; 35: 605-22

22. Candy B, Jones L, Drake R, Leurent B, King M. Interventions for supporting informal caregivers of patients in the terminal phase of a disease. Cochrane Database Syst Rev 2011: CD007617

23. Plonk WM, Jr., Arnold RM. Terminal care: the last weeks of life. J Palliat Med 2005; 8: 1042-54

24. Andershed B. Relatives in end-of-life care--part 1: a systematic review of the literature the five last years, January 1999-February 2004. J Clin Nurs 2006; 15: 1158-69

25. Ostlund U, Brown H, Johnston B. Dignity conserving care at end-of-life: a narrative review. Eur J Oncol Nurs 2012; 16: 353-67.

26. Hudson PL, Remedios C, Thomas K. A systematic review of psychosocial interventions for family carers of palliative care patients. BMC Palliat Care 2010; 9: 17

27. Jennings AL, Davies AN, Higgins JPT, Anzures-Cabrera J, Broadley Karen E. Opioids for the palliation of breathlessness in advanced disease and terminal illness. Cochrane Database of Systematic Reviews 2012: CD002066

28. Mahler DA, Selecky PA, Harrod CG, Benditt JO, Carrieri-Kohlman V, Curtis JR, Manning HL, Mularski RA, Varkey B, et al. American College of Chest Physicians consensus statement on the management of dyspnea in patients with advanced lung or heart disease. Chest 2010; 137: 674-91

29. Gallagher R, Roberts D. A systematic review of oxygen and airflow effect on relief of dyspnea at rest in patients with advanced disease of any cause. J Pain Palliat Care Pharmacother 2004; 18: 3-15.

30. Booth S, Wade R, Johnson M, Kite S, Swannick M, Anderson H. The use of oxygen in the palliation of breathlessness. A report of the expert working group of the Scientific Committee of the Association of Palliative Medicine. Respir Med 2004; 98: 66-77

31. Simon ST, Higginson IJ, Booth S, Harding R, Bausewein C. Benzodiazepines for the relief of breathlessness in advanced malignant and non-malignant diseases in adults. Cochrane Database Syst Rev 2010: CD007354

32. Ujeyl M, Muller-Oerlinghausen B. [Antidepressants for treatment of depression in palliative patients : a systematic literature review]. Schmerz 2012; 26: 523-36
