# Supplementary material for: How should we manage information needs, family anxiety, depression, and breathlessness for those affected by advanced disease: development of a Clinical Decision Support Tool using a Delphi design
Source: BMC Med. 2015 Oct 13;13:263. doi: 10.1186/s12916-015-0449-6 (PMC4604738; doi:10.1186/s12916-015-0449-6)
Supplement: Additional file 3: — Demographics participants. (DOCX 18 kb) [file 12916_2015_449_MOESM3_ESM.docx]

## Additional File 3 – demographics participants online Delphi study

|  | **Round 1** | **Round 2** |
| --- | --- | --- |
|  | **N=25** | **N=23** |
| **Gender** |  |  |
| Male | 12 | 13 |
| Female | 13 | 10 |
|  |  |  |
| **Age category** |  |  |
| 20-30 | 1 | 1 |
| 31-40 | 4 | 3 |
| 41-50 | 9 | 7 |
| 51-60 | 6 | 8 |
| 61-70 | 4 | 2 |
| >70 | 1 | 2 |
|  |  |  |
| **Country of residence** |  |  |
| Belgium | 1 | 2 |
| Germany | 3 | 3 |
| Italy | 1 | 1 |
| Netherlands | 4 | 2 |
| South Africa | 1 | 1 |
| Sweden | 2 | 2 |
| US | 1 | 1 |
| UK | 10 | 8 |
| Greece | 1 | 0 |
| Switzerland |  | 1 |
| Missing | 1 | 2 |
|  |  |  |
| **Profession** |  |  |
| Palliative care physician | 9 | 9 |
| General Practitioner | 2 | 2 |
| Nurse | 4 | 3 |
| Social worker | 1 | 2 |
| Researcher | 13 | 12 |
| Patient Representative | 3 | 3 |
| MD | 1 | 0 |
| Allied Health Profession | 1 | 1 |
| Oncologist |  | 1 |
| Missing | 1 | 0 |
|  |  |  |
| **Work setting (multiple answers may apply)** |  |  |
| Hospital | 15 | 8 |
| Hospice | 3 | 2 |
| Care Homes | 0 | 1 |
| Community | 6 | 4 |
| Palliative care team | 9 | 8 |
| Patient Representative | 2 | 3 |
| Consultant | 1 | 1 |
| AHP |  | 1 |
| General practice / family practice | 1 | 1 |
| University / Research | 2 | 2 |
| Missing | 3 | 0 |
|  |  |  |
| **Years of experience in palliative care** |  |  |
| 0 | 8 | 6 |
| 1-5 | 3 | 2 |
| 6-10 | 4 | 2 |
| 11-15 | 5 | 6 |
| 16-20 | 2 | 5 |
| 21-25 | 1 | 1 |
| 25-30 | 2 | 1 |
|  |  |  |
|  |  |  |
| **Own rated expertise/skills in palliative car** |  |  |
| None | 2 | 0 |
| Somewhat | 3 | 4 |
| Quite a lot | 7 | 7 |
| Experts | 9 | 10 |
| Other – husband died at home. Participant was main carer | 1 | 1 |
| Other – especially expert about GP-patient communication in palliative care | 1 | 0 |
| Other – responses were discussed with palliative care team before submission | 1 | 0 |
| Other – not working in practice | 1 | 0 |
| Missing | 0 | 1 |
|  |  |  |
| **Do you know the POS** |  |  |
| Yes | 20 | 18 |
| No | 5 | 4 |
| Missing |  | 1 |
|  |  |  |
| **Have you used the POS in clinical care?** |  |  |
| Yes – using it at the moment | 4 | 3 |
| Yes – but stopped using them | 2 | 3 |
| No – never considered | 5 | 4 |
| No – considered but never used | 4 | 5 |
| Not applicable – I do not work in clinical care | 9 | 6 |
| Missing | 1 | 2 |
|  |  |  |
| **Have you used the POS in research?** |  |  |
| Yes – using it at the moment | 7 | 8 |
| Yes – but stopped using them | 3 | 3 |
| No – never considered | 4 | 3 |
| No – considered but never used | 6 | 4 |
| Not applicable – I do not work in research | 5 | 4 |
| Missing | 0 | 1 |
