# Supplementary material for: How should we manage information needs, family anxiety, depression, and breathlessness for those affected by advanced disease: development of a Clinical Decision Support Tool using a Delphi design
Source: BMC Med. 2015 Oct 13;13:263. doi: 10.1186/s12916-015-0449-6 (PMC4604738; doi:10.1186/s12916-015-0449-6)
Supplement: Additional file 4: — Short manual of Clinical Decision Support Tools. (DOCX 4651 kb) [file 12916_2015_449_MOESM4_ESM.docx]

| Short manual:Clinical Decision Support Tool for the interpretation of and response to Palliative care Outcome Scale (POS) scores for:information needsfamily anxietydepressionbreathlessness |
| --- |
| Developed on behalf of EUROIMPACT (European Intersectorial and Multidisciplinary Palliative Care Research Training). EURO IMPACT is funded by the European Union Seventh Framework Programme (FP7/2007-2013, under grant agreement nr [264697]). |
|  |
|  |

## Background and scope

The Palliative (or Patient) care Outcome Scale (POS) is a multidimensional outcome measure which can be used in clinical palliative care to assess patients’ symptoms and concerns and monitor changes in these over time. It assesses physical symptoms, emotional, psychological and spiritual concerns, and needs for information and support. It is brief (<10 minutes to complete), widely validated, able to transfer across settings, has good responsiveness to change and has been translated and/or culturally adapted and revalidated in many different languages and cultures. The core POS is part of a family of POS measures, including the POS-S (focused on physical symptoms), the APCA African POS (for the African setting) and the IPOS (integrating the POS, POS-S and APCA African POS). More information can be found at <http://pos-pal.org/>.

Despite POS being widely used, there is still a need for more support on how to use the measure and respond clinically to symptoms identified. Clinicians encounter the most difficulty with responding to non-physical symptoms, i.e. depression, information needs, family anxiety and to the symptom of breathlessness. We therefore developed a Clinical Decision Support Tool (CDST), consisting of evidence-based and expert-endorsed recommendations to help doctors, nurses and other clinicians make decisions on how to respond to different levels of these problems and symptoms as measured by the POS. This CDST can be used to assist clinical responses to specific POS scores. It can be used for all POS family of measures (e.g. IPOS, APCA African POS), by all practitioners working with palliative care populations. It is not intended to be prescriptive; it aims to help practitioners think through the best decisions for these difficult and complex problems. It should be used alongside skilled individual clinical assessments and knowledge, taking into account patients’ and families’ individual preferences, circumstances and available resources. The CDST should be used as a starting point to achieve high quality person-centered care and good clinical practice.

The quality of the evidence is based on an ABCD framework (an adapted GRADE framework) with A (reflecting ‘high’ in GRADE; e.g. meta-analysis, SR of RCT, RCT), B (reflecting ‘moderate’ in GRADE; e.g. cohort studies, case-control studies), C (reflecting ‘low’ in GRADE; e.g. retrospective, poor quality cohort studies) and D (reflecting ‘very low’ in GRADE’; qualitative studies, expert opinion) quality evidence for recommendations.

**More information**

For a more detailed overview of this project, please consult the full manual:

van Vliet LM, Harding R, Bausewein C, Payne S, Higginson IJ. Clinical Decision Support Tool for the interpretation of and response to Palliative care Outcome Scale (POS) scores for information needs, family anxiety, depression and breathlessness: Full manual including references and evidence. London: Department of Palliative Care, Policy & Rehabilitation; 2015

*Note that the quality of research evidence should be interpreted with caution. The provided research evidence indicates the nature of the research designs (or the ratings already assigned by different sources) which have assessed the studies in this field. Where the quality is low it implies that there have been few comparative studies, and that there is an absence of evidence either supporting or not supporting the approach. However, this does not indicate the strength of the recommendation.

**We would like to refer to the Vitaltalk website for more resources about responding to patients’ information needs and related communication issues http://www.vitaltalk.org/

**Information needs**

**Over the past 3 days, how much information have you and your family and friends been given?**

**Offer, depending on resources, a care meeting with the patient, family (members should be agreed by patient) and health care providers to discuss the condition, course of illness, individuals’ preferences, treatment options and plan**. Care meetings can promote communication, trust, realistic hope, increase clinicians’ knowledge of the patient and decrease stress by reviewing realistic goals.

(C)

Information given on request but would have liked more (2) + Very little given and some questions were avoided (3) + None at all – when we wanted information (4)

*All of above recommendations +*

**Always assess patients’ preferences for information,** including the specific content and extent of information that is preferred (e.g. ask “are you the sort of person who likes to know everything about their disease”). Provided information should be based on these preferences

(B)

**Conduct a ‘cultural’ assessment** (assess the cultural context), including the preferences for information disclosure and decision-making of an individual. Be aware that not all ethnic groups prefer to be directly informed of a life-threatening diagnosis; sometimes only the family wishes to be informed (or involved in decision making). Patients’ and their families’ wishes not to take part in decisions should be respected.

(C)

**Relevant information must be provided – if possible – in a quiet, comfortable place with privacy and without interruptions.**

(D)

**Provide – individually tailored - information honestly, sensitively, with margin for and balance with hope**. Hope comprises more than hope for a cure or life prolongation, but also includes focus on achieving something or the way that remaining time is spent.

(D)

**Provide clear information and assess (in a caring way) the patient’s understanding** of the illness and of the provided information

(D)

**Verbal face-to-face information can be accompanied by other methods** such as written information (based on individual preferences).

(B)

**Always show an empathic attitude.** Important behaviours include: a willingness to listen, the use of eye-contact, responses to (non)verbal cues and acknowledgement of the patient as an individual

(D)

Full information or as much as they wanted (0) + Information given but hard to understand (1)

*Please consult the following guidelines for more detailed information about recommended pharmacological interventions.

1) Wiseman et al 2013. Chronic refractory dyspnoea. Evidence based management. Australian Family Physician, 42;.3, 137-140: <http://www.racgp.org.au/afp/2013/march/chronic-refractory-dyspnoea/>

2) Palliative Care Guidelines NHS Scotland: <http://www.palliativecareguidelines.scot.nhs.uk/documents/breathlessnessfinal.pdf>

When breathlessness is strongly associated with anxiety, we would like to refer to the following guideline which provides in-depth guidance on how to respond to anxiety in palliative care: <http://www.eperc.mcw.edu/EPERC/FastFactsIndex/ff_145.htm>

**Note that the quality of research evidence should be interpret with caution. The provided research evidence indicates the nature of the research designs (or the ratings already assigned by different sources) which have assessed the studies in this field. Where the quality is low it implies that there have been few comparative studies, and that there is an absence of evidence either supporting or not supporting the approach. However, this does not indicate the strength of the recommendation.

*Note that the quality of research evidence should be interpreted with caution. The provided research evidence indicates the nature of the research designs (or the ratings already assigned by different sources) which have assessed the studies in this field. Where the quality is low it implies that there have been few comparative studies, and that there is an absence of evidence either supporting or not supporting the approach. However, this does not indicate the strength of the recommendation.

**The (most vulnerable) family should after full assessment and discussion be offered referral to intensive support services.**

(D)

(_)

Most of the time (3) / Yes, always (4)

*All of above recommendations +*

Not at all (0)

**Family anxiety**

**Over the past 3 days, have any of your family or friends been anxious or worried about you?**

**In the social/psychological needs assessment, it is important to assess caregiver’s own health status, other commitments and ability to continue the caring role**. Caregivers can be older and/or may have health problems of their own.

(A)

**A social and psychological needs assessment should be offered (to the patient and family) to determine current state and risk of poor psychological health and grief**. An assessment can include the creation of a genogram/ecomap to cover the family structure and dynamics, and can assess families’ lines of communication, geographic location, needs and goals (e.g. counselling needs, needs for equipment), and strength and vulnerabilities (e.g. finances, prior experiences, sexuality/intimacy, caregiver availability).

(A)

Occasionally (1)

*All of above recommendations +*

Sometimes (2)

*All of above recommendations +*

**Following assessment, interventions should be (promptly) planned,** which are focused on carer’s needs, goals and preferences and based on best evidence.

(A)

**Start by exploring with the patient and family (if agreed by patient) if they are anxious and if so what is causing this anxiety.**

(D)

**A family conference should be offered (with patient consent) to exchange information, assess needs, provide emotional support and create a care plan** (if feasible and resources allow).

(A)

**Practical help to remove barriers to care should be provided,** depending on resources and needs, such as transportation for appointments, home care, 24-hour medical advice, and applying for benefits.

(no available evidence)

**Optimize care (pain/symptom management) of the patient**

(B)

**The family should be offered - depending on needs, feasibility and resources - the opportunity to sometimes withdraw from the caregiving situation** (i.e. get some mental rest/respite care) or get suggestions on how to organise this themselves (e.g. with help from family/friends)

(D)

**Good communication to keep the family informed should be provided** (with patient consent). Information should be adjusted (i.e. tailored) to the family’s needs and be provided in comprehensible language based on the family’s understanding of the illness (e.g. say “the cancer has spread” instead of “the cancer has metastasized”).

(D)

**Emotional support should be provided.** This is characterized by listening, empathy, caring, and a continued trusting relationship in which relatives can express their concerns and – if appreciated – feel a valued part of the team

(D)

**(Psycho) Education should be offered** (i.e. teaching, explanation). Depending on the situation, family wishes and understanding, this could include information on how to manage the care of a loved one and on the signs of approaching death. Families often want to be present and to help, and this involvement can improve the relationship between staff and family.

(A)

**Support should be provided with respect for and facilitation of cultural, religious and social practices and traditions.**

(no available evidence)

*Please consult the following guidelines for more detailed information about recommended pharmacological interventions.

1) Wiseman et al 2013. Chronic refractory dyspnoea. Evidence based management. Australian Family Physician, 42;.3, 137-140: <http://www.racgp.org.au/afp/2013/march/chronic-refractory-dyspnoea/>

2) Palliative Care Guidelines NHS Scotland: <http://www.palliativecareguidelines.scot.nhs.uk/documents/breathlessnessfinal.pdf>

When breathlessness is strongly associated with anxiety, we would like to refer to the following guideline which provides in-depth guidance on how to respond to anxiety in palliative care: <http://www.eperc.mcw.edu/EPERC/FastFactsIndex/ff_145.htm>

**Note that the quality of research evidence should be interpret with caution. The provided research evidence indicates the nature of the research designs (or the ratings already assigned by different sources) which have assessed the studies in this field. Where the quality is low it implies that there have been few comparative studies, and that there is an absence of evidence either supporting or not supporting the approach. However, this does not indicate the strength of the recommendation.

No, not at all (0) + Occasionally (1)

Most of the time (3) + Yes, all the time (4)

*All of above recommendations +*

**Enquire actively about patients’ concerns/feelings and provide emotional support** (e.g. provide a listening ear), if appreciated.

(B)

**Communicate openly with patients and provide information (on all topics) in accordance with their preferences**; e.g. determine their needs for information (they can change over time) and discuss information in appropriate language.

(B)

Sometimes (2)

*All of above recommendations +*

**Focus on cognitive/affective symptoms in detecting depression alongside physical symptoms**, as the latter might be caused by the physical disease or the medical treatment. Examples of cognitive/affective symptoms are: dysphoric mood, excessive hopelessness, social withdrawal, suicidal thoughts. Examples of physical symptoms are: weight loss, insomnia, loss of energy, fatigue. Focus on the course of these physical symptoms in detecting depression and consider what triggered similar symptoms before.

(B)

**Inform patients about sources for support** (e.g. community groups).

(B)

**Depression**

**POS Question: Over the past 3 days, have you been feeling depressed**

*Note that the quality of research evidence should be interpreted with caution. The provided research evidence indicates the nature of the research designs (or the ratings already assigned by different sources) which have assessed the studies in this field. Where the quality is low it implies that there have been few comparative studies, and that there is an absence of evidence either supporting or not supporting the approach. However, this does not indicate the strength of the recommendation.

******Please consult the following guidelines for more detailed information about recommended antidepressants:

1) Rayner et al 2010. The management of depression in palliative care: European Clinical Guidelines. London: Department of Palliative Care, Policy & Rehabilitation/European Palliative Care Research Collaborative): <http://www.epcrc.org/getpublication2.php?id=6VW4bQY9JujQVGSItDs6>

2) Palliative Care Guidelines NHS Scotland: <http://www.palliativecareguidelines.scot.nhs.uk/documents/depressionfinal.pdf>

**Conduct a psychosocial assessment** (to differentiate between low mood and depression); screen – if feasible – for depression with measures such as the Brief Edinburgh Depression Scale, PHQ-9 or HADS. Subsequently, diagnose depression with criteria such as these of the DSM-IV or ICD-10.

(D)

**Refer – depending on resources – patients to specialist palliative care service**s for improved symptom control and psychosocial support. Addressing problems which are physical (e.g. pain), psychological (e.g. lack of information), social (e.g. family conflict) or spiritual (e.g. existential questions) may alleviate depressive symptoms.

(A)

**Offer psychological therapy (depending on assessment and resources).** Consider factors such as time (treatment might need to be short because of life-expectancy) and patient preferences in choosing the therapy. Therapies with proven effectiveness include: CBT and psychotherapy (n.b. a diagnosis of depression is needed for these interventions).

(A)

**Offer antidepressants after careful assessment/diagnosis and consideration of non-drug interventions.** An open discussion of options should be held in which antidepressants are not provided as ‘fixed solutions’. Consider factors such as life expectancy, side effects, risk of suicide, possible interactions and contraindications, and patient and clinician preferences in choosing the antidepressant. Therapies with proven effectiveness include: SSRI’s, mirtazapine, TCA’s**.

(A)

**

**Ensure treatment for any underlying causes is optimised.**

(D)

Moderately (2)

*All of above recommendations +*

**A physical examination and complete holistic history should be done** – early on – to determine factors that likely have influenced the severity of symptoms.

(C)

Severely (3)

*All of above recommendations +*

**Reversible causes of breathlessness should be treated if indicated/appropriate and the patient wants this**. Examples include: heart failure, exacerbations of COPD, cardiac arrhythmias, anaemia, pleural or pericardial haemorrhage, bronchial infection, pulmonary embolism, superior vena cava syndrome, pleural effusion, pain, and depression.

(C)

**Non-pharmacological evidence-based interventions should be used** to treat breathlessness (if patient is able to participate).

(A)

**The offer to use walking aids** (following physical assessment)

(B)

**Education and support around the pacing of daily tasks and encouraging physical activity,** tailored to individual.

(D)

**Education and support around breathing control/management techniques** e.g. active cycle of breathing/ pursed lip breathing, (taking patient preference into account).

(B)

**Pharmacological evidence-based interventions should be offered to treat breathlessness in conjunction with non-pharmacological** interventions and carefully monitored**.

(A)

**The use of a fan.**

(no available evidence**)**

**Psychosocial support appropriate to situation,** e.g. coping, goal-setting, distraction /relaxation, and meditation /mindfulness.

(No available evidence)

Slightly (1)

*Please consult the following guidelines for more detailed information about recommended pharmacological interventions.

1) Wiseman et al 2013. Chronic refractory dyspnoea. Evidence based management. Australian Family Physician, 42;.3, 137-140: <http://www.racgp.org.au/afp/2013/march/chronic-refractory-dyspnoea/>

2) Palliative Care Guidelines NHS Scotland: <http://www.palliativecareguidelines.scot.nhs.uk/documents/breathlessnessfinal.pdf>

When breathlessness is strongly associated with anxiety, we would like to refer to the following guideline which provides in-depth guidance on how to respond to anxiety in palliative care: <http://www.eperc.mcw.edu/EPERC/FastFactsIndex/ff_145.htm>

**Note that the quality of research evidence should be interpret with caution. The provided research evidence indicates the nature of the research designs (or the ratings already assigned by different sources) which have assessed the studies in this field. Where the quality is low it implies that there have been few comparative studies, and that there is an absence of evidence either supporting or not supporting the approach. However, this does not indicate the strength of the recommendation.

**Breathlessness**

**how do you feel the symptom ‘Shortness of breath’ has affected you and how you have been feeling over the past week**

*Note that the quality of research evidence should be interpreted with caution. The provided research evidence indicates the nature of the research designs (or the ratings already assigned by different sources) which have assessed the studies in this field. Where the quality is low it implies that there have been few comparative studies, and that there is an absence of evidence either supporting or not supporting the approach. However, this does not indicate the strength of the recommendation.

**Please consult the following guidelines for more detailed information about recommended pharmacological interventions.

1) Wiseman et al 2013. Chronic refractory dyspnoea. Evidence based management. Australian Family Physician, 42;.3, 137-140: <http://www.racgp.org.au/afp/2013/march/chronic-refractory-dyspnoea/>

2) Palliative Care Guidelines NHS Scotland: <http://www.palliativecareguidelines.scot.nhs.uk/documents/breathlessnessfinal.pdf>

When breathlessness is strongly associated with anxiety, we would like to refer to the following guideline which provides in-depth guidance on how to respond to anxiety in palliative care: <http://www.eperc.mcw.edu/EPERC/FastFactsIndex/ff_145.htm>

***We would like to refer to the Cicely Saunders Institute’s’ Breathlessness Intervention Service for resources on managing breathlessness: http://www.kcl.ac.uk/lsm/research/divisions/cicelysaunders/research/symptom/breathlessness.aspx

Overwhelmingly (4)

*All of above recommendations +*

**Chest wall vibration** (a non-invasive therapy which aims to stimulate respiratory muscles which may reduce breathlessness).

(B)

(_)

**Provide oxygen for patients who are hypoxemic at rest or during minimal activity and after careful thought, assessment and individualisation.**

(C)

**Other medications might be useful as well as second-line drugs and could be tested in a therapeutic trial** (within a patient); including benzodiazepine (especially if associated with anxiety/panic), promethazine, corticosteroids, steroids, bronchodilators and SSRI’s.

(no available evidence)

**Neuromuscular electrical stimulation** (NMES – non-invasive therapy to improve peripheral muscle strength and exercise capacity which may impact favourably on breathlessness), **if patients cannot exercise themselves** (mainly in non-cancer settings, depending on cause)

(A)

**Opioids via oral (mouth) or parenteral (drip) route, using a sustained release (long-acting) low dose.**

(A)

**Funding**

European Intersectorial and Multidisciplinary Palliative Care Research Training (EURO IMPACT) is funded by the European Union Seventh Framework Programme (FP7/2007-2013, under grant agreement number [264697]). EURO IMPACT aims to develop a multidisciplinary, multi-professional and inter-sectorial educational and research training framework for palliative care research in Europe. EURO IMPACT is coordinated by Prof. Luc Deliens and Prof. Lieve Van den Block of the End-of-Life Care Research Group, Ghent University and Vrije Universiteit Brussel, Brussels, Belgium. Other partners are VU University Medical Center, EMGO Institute for Health and Care Research, Amsterdam, the Netherlands; King’s College London, Cicely Saunders Institute, London, Cicely Saunders International, London, and International Observatory on End-of-Life Care, Lancaster University, Lancaster, United Kingdom; Norwegian University of Science and Technology, and EAPC Research Network, Trondheim, Norway; Regional Palliative Care Network, IRCCS AOU San Martino–IST, Genoa, and Cancer Research and Prevention Institute, Florence, Italy; EUGMS European Union Geriatric Medicine Society, Geneva, Switzerland; Springer Science and Business Media, Houten, the Netherlands.

This booklet is also supported in part by Cicely Saunders International, and presents independent research funded by the UK National Institute for Health Research (NIHR) Senior Investigator Award (Higginson), the Collaboration for Leadership in Applied Health Research and Care (CLAHRC) South and the UK National Institute for Health Research (NIHR) funded C-CHANGE project (project number RP-PG-1210-12015).The Collaboration for Leadership in Applied Health Research and Care (CLAHRC) South London is part of the National Institute for Health Research (NIHR), and is a partnership between King’s Health Partners, St. George’s, University London, and St George’s Healthcare NHS Trust.

The views and opinions expressed by authors are those of the authors and do not necessarily reflect those of the NHS, MRC, CCF, NETSCC, the UK NIHR Programme Grants for Applied Research programme or the UK Department of Health.
